# Cognitive interviewing of the older adults with cancer – depression scale (OAC-D): A patient-reported outcome (PRO)

**DOI:** 10.1017/S1478951525000355

**Published:** 2025-04-22

**Authors:** Rebecca M. Saracino, Laura C. Polacek, Rebecca Tutino, Hayley Pessin, Kathleen A. Lynch, Thomas M. Atkinson, Christian J. Nelson

**Affiliations:** Department of Psychiatry & Behavioral Sciences, Memorial Sloan Kettering Cancer Center, New York, NY, USA

**Keywords:** Depression, cancer, geriatric assessment, aging, screening

## Abstract

**Objectives:**

This study aimed to refine the content of a new patient-reported outcome (PRO) measure via cognitive interviewing techniques to assess the unique presentation of depressive symptoms in older adults with cancer (OACs).

**Methods:**

OACs (≥ 70years) with a history of a depressive disorder were administered a draft measure of the *Older Adults with Cancer – Depression (OAC-D) Scale*, then participated in a semi-structured cognitive interview to provide feedback on the appropriateness, comprehensibility, and overall acceptability of measure. Interviews were audio-recorded and transcribed, and qualitative methods guided revision of scale content and structure.

**Results:**

OACs (*N* = 10) with a range of cancer diagnoses completed cognitive interviews. Participants felt that the draft measure took a reasonable amount of time to answer and was easily understandable. They favored having item prompts and response anchors repeated with each item for ease of completion, and they helped identify phrasing and wording of key terms consistent with the authors’ intended constructs. From this feedback, a revised version of the OAC-D was created.

**Significance of results:**

The *OAC-D Scale* is the first PRO developed specifically for use with OACs. The use of expert and patient input and rigorous cognitive interviewing methods provides a conceptually accurate means of assessing the unique symptom experience of OACs with depression.

## Background

Older adults with cancer (OACs), which includes those aged 65 and older, account for nearly 60% of new cancer diagnoses (American Cancer Society 2024; Bluethmann et al. 2016). According to the 2020 U.S. Census, there are over 55.8 million older adults living domestically; this number is expected to climb to 80 million by 2040. Cancer in older age is often associated with significant mental health concerns such as depression, with a prevalence in OACs as high as 25% (Clark et al. 2016; Frazzetto et al. 2012; Parajuli et al. 2021). Comorbid cancer and depression can lead to declines in functioning and health-related quality of life, increased pain and fatigue, treatment interference and discontinuation, higher healthcare costs, and in the extreme, premature mortality and suicide (DiMatteo et al. 2000; Erlangsen et al. 2015; Wang et al. 2020). Despite the importance of screening for depression among OACs, there are many challenges to accurate identification of depressive symptoms in both older adults and individuals with cancer, including symptom overlap with cancer treatment side effects and a tendency for older adults to not report classic symptoms of depression such as depressed mood. As a result, existing measures of depression that primarily rely on Diagnostic and Statistical Manual of Mental Disorders (DSM) criteria may not be conceptually accurate for capturing the experiences of OACs with depression.

Our systematic review examined the development and psychometric properties of the most utilized depression screeners in geriatric, cancer, and geriatric cancer populations using the U.S. Food and Drug Administration (FDA) Patient-Reported Outcomes (PROs) Guidance as the framework for evaluation (Nelson et al. 2010). We found that while several measures had been retrospectively evaluated for use with older adults and patients with cancer, separately, none had examined their utility for use with OACs, and certainly none had been developed specifically for use with OACs. Moreover, none of these involved patient input or the standard process of cognitive interviewing to develop content validity throughout their development (Patrick et al. 2011; U.S. Department of Health and Human Services 2009). Given the lack of psychometric validity to support for the use of these measures with OACs, we conducted a study to examine their psychometric properties in this population (Saracino et al. 2017). Participants were 201 OACs on cancer treatment, ≥ 70years, recruited from outpatient clinics at MSK. They completed the GDS-Short Form (GDS-SF; Yesavage 1988), HADS-Depression (HADS-D; Zigmond and Snaith 1983), and Center for Epidemiological Studies Depression Scale-Revised (CESD-R; Van Dam and Earleywine 2011) and were interviewed using the depression module of the Structured Clinical Interview for DSM disorders (SCID; First 2014) as the gold standard comparison. We found that each measure had adequate internal consistency and predicted depression on the SCID greater than chance. However, using the published cut-off scores produced grossly inadequate sensitivity, with the potential to “miss” anywhere from 33 to 83% of OACs who are indeed experiencing MDD. We concluded by recommending lower cut-off scores (i.e., 4 vs. 5 on the GDS-SF, 6 vs. 8 on the HADS-D, and 15 vs.16 on the CESD-R). While using lower cut-off scores is one option for depression screening in OACs it may result in more false positives and associated burden on limited clinical resources. Furthermore, the cumulative lack of patient and expert input during the development of these measures and their overall poor psychometric performance in OACs amplifies the limitations of existing tools and suggests that starting from the ground up to develop a new PRO for this unique patient population is warranted.

As an initial step in this process, we implemented a semi-structured survey of internationally recognized experts in geriatric psychiatry and oncology (*N* = 8; Saracino et al. 2024). Using an inductive qualitative approach to minimize assumptions and the circularity of relying on DSM criteria, we queried experts about the signs and symptoms they observe in depressed versus non-depressed OACs. Thematic content analyses revealed key themes pertaining to (1) key indicators of depression in OACs and (2) unique considerations for evaluating depression in OACs. Experts described the most salient features of depression in OACs as anhedonia, loneliness and social withdrawal, a sense of malaise, tearfulness and crying, loss of meaning and purpose and a sense of futility, and negative ruminations and hopelessness. As suggested in our previous studies (Nelson et al. 2010; Saracino et al. 2017), these signs and symptoms are nuanced beyond DSM criteria. Only anhedonia emerged as retaining its clinical utility among DSM criteria.

The overarching conclusion of both our systematic narrative review and expert interview study was that there was a clear opportunity to improve our conceptualization and assessment of depression for OACs through development of a new PRO not restricted to DSM criteria. Next, we utilized the refined conceptual framework (Saracino et al. 2023) of depression (i.e., based on existing literature and expert interviews) in qualitative interviews with 26 older adults (≥70 years) with a history of cancer (depressed *n* = 13; non-depressed *n* = 13). Thematic analyses revealed four major (i.e., Interest and Enjoyment (Anhedonia), Loneliness/Social Withdrawal, Lack of Meaning/Purpose, Lack of Usefulness/Feeling Like a Burden) and four minor themes (i.e., Depressed Mood, Regret and Guilt, Attitudes Towards Treatment, Attitudes Towards Physical Symptoms/Limitations). Six of these themes departed from DSM symptoms. Participants’ insights were remarkably consistent with the concepts described by our experts and formed the key domains of the draft measure, reinforcing the refined conceptual framework of depression.

With these constructs defined, our team generated a list of 37 candidate items for a draft measure, the *Older Adults with Cancer – Depression (OAC-D)* scale. We developed four versions of the measure with varying formats and response options. In the present study, we conducted cognitive interviews of the draft measure. Cognitive interviewing is an important, but sometimes overlooked, component of PRO development. The method ensures the understandability and user-friendliness of questionnaire items. From a phenomenological perspective, these interviews are a form of “member checking,” confirming that the OAC-D items accurately captured their experiences with depression (Tuffour 2017). Thus, the aim of the current study was to obtain patient-level feedback on the appropriateness, comprehensibility, and overall acceptability of the draft OAC-D items.

## Methods

### Procedures

This study was approved by the Memorial Sloan Kettering Cancer Center (MSK) Institutional Review Boards (#14-101). Potential participants were identified by a review of MSK Counseling Center clinic lists. Recruitment was conducted by a trained research assistant (RT). Inclusion criteria were (1) ≥70 years old, (2) history or current diagnosis of cancer, and (3) history of depression, dysthymia, or adjustment disorder with depressed mood. Individuals were excluded if they (1) scored ≥11 on the Blessed Orientation-Memory-Concentration Test (BOMC; Katzman et al. 1983) or (2) exhibited severe psychopathology or cognitive impairment likely to interfere with the participation or completion of the protocol or ability to provide meaningful information. Eligibility was confirmed through verification of the electronic medical record.

Participants were approached in person or contacted by telephone and invited to learn about the research study. Those who were interested in participation completed a cognitive screener (i.e., BOMC) to confirm eligibility. Those who were eligible and interested provided informed consent before completing a demographic questionnaire and scheduling their study interview.

### Cognitive interviews

Under guidance from the Qualitative Methods Specialist (QMS; KL), a semi-structured cognitive interview guide was developed to include verbal probes to elicit feedback about the OAC-D questionnaire items and response options (e.g., structure, format), recall period, and item content (e.g., appropriateness, acceptability, comprehension of item wording). Retrospective probing was utilized such that participants were presented with four versions of the questionnaire (i.e., item content was consistent across versions, but response option layout varied) and asked to select the one that was most appealing to them. Next, they completed the questionnaire in the presence of the study interviewer (i.e., graduate level doctoral students in clinical psychology, RS, LP) using paper and pen. They were then asked about their responses during cognitive interviews, which included describing item concepts and definitions in their own words (e.g., “what does the phrase ‘feeling down’ mean to you?”).

One round of cognitive interviewing was planned, with opportunity for additional rounds if consensus was not clear after the initial round. A sample size of 10–15 was selected for the initial round, with a plan to review the interview data after the first 10 participants to determine if additional interviews were necessary to reach content saturation (i.e., the point at which no additional information suggesting questionnaire modification emerges; Guest et al. 2006). All interviews were conducted in person, in a single session, in private rooms at the MSK Counseling Center. Participants received $20.00 as compensation for participation.

### Analysis

All interviews were audio-recorded and transcribed. Interview transcripts and interviewer observational notes taken during the cognitive interview sessions were utilized to create a structured summary of each interview. Summaries included response format option preference, items that were marked as difficult and corresponding participant explanations or alternative wording suggestions, comprehension and meaning assigned to different item wording, perceived difficulty of completing the questionnaire, acceptability of questionnaire length, and free response comments elicited at the end of the interviews (e.g., *do you have any additional advice on how we can improve this questionnaire?*). An overall summary chart of participant feedback was then compiled from individual interview summaries. The summary was then systematically reviewed by the study team as a group (led by QMS KL) to reach consensus on the revised structure and content of the scale. While items with the most comments were discussed first, all items on the scale were reviewed regardless of how many concerns were identified to improve the measure before piloting it with a larger sample (Willis GB 2005). Based on the summary discussion after the first 10 interviews, the group determined that content saturation was achieved, and it was decided that no additional cognitive interview rounds were necessary at this phase of the research.

## Results

### Participant demographics

Participants were 10 older adults with a history of cancer. The sample was roughly evenly split by gender (60% male; *n* = 6). with a mean age of 77 (range: 71–88). All participants identified as non-Hispanic white race. Cancer history was heterogeneous with pancreatic, prostate, bladder, and uterine cancers represented in the sample.

### Overall impressions and satisfaction

Participants unanimously reported that there were no challenges to completing the questionnaire overall, nor challenges to completing the cognitive interviewing. Participants described most of the items as “easy” to answer. They also felt that the amount of time it took to complete the questionnaire was reasonable. Overall participants felt the questionnaire was comprehensive and did not report the need to add additional items, they agreed that the questionnaire adequately assessed their experiences with depression.

### Response prompt, format, anchors, and recall period

Participants reported satisfaction with the prompt “*I noticed that*” and commented on its broad applicability (e.g., “a wider scope of experience than just mood”). Most participants selected format D. In this format, the prompt “I noticed that” and response anchors (i.e., Never, Rarely, Sometimes, Often, Always), are repeated with each item rather than being listed only once at the top of the questionnaire. Participants felt this option was clearer and easier to read than the others. Half of participants endorsed slight difficulty when deciding between response anchors. However, they felt this was due to thinking through their own experience rather than a problem with the anchors themselves. They did not find any of the options confusing or difficult to understand. Given the clear consensus for format D, the research team selected it as appropriate for the final measure.

### Item-level feedback

*Items marked as difficult.* Across all 37 items, three participants marked an item as “difficult” (i.e., item 11, 18, and 30). Upon probing, participants reported that the items were not difficult to understand, per say, but rather they did not personally identify with the item content.

*Participant perceptions and understanding of item content.* Participants were asked about the specific personal meaning of several constructs to confirm wording. For example, preferences between “valuable” or “worthwhile,” and “irritated” or “irritable.” A summary of participants’ feedback is described in Table 1. Participants’ personal descriptions or definitions of terms were consistent with the authors’ intended constructs. Participants were also specifically queried about their understanding and preferences among three item pairs to facilitate item reduction (Table 2).

**Table 1.** Cognitive interview querying of selected OAC-D terms

Selected phrases	Illustrative participant descriptions	Consistent with intended meaning (yes/no)
Things	*“Behaviors, things that I’ve done, more than just activities.”*	Yes
Disconnected from other people	*“Less interested in what they’re talking about; being quiet at dinner and not entering the conversation.”*	Yes
Isolated	*“Separated from – communication, physically, emotionally.”*	Yes
Feeling down	*“Means depressed, but it is good you did not use the word depressed, it is too harsh; this has less stigma.”*	Yes
Tearful	*“Easily saddened by things, emotionally touched by things, sometimes actually tearing up.”*	Yes
Resentful	*“Something occurred that is hurtful and it doesn’t have to be that way.”*	Yes
Hassle	*“There are activities that are burdensome and it doesn’t seem to be a good reason for the activity to occur the way it is; inefficient.”*	Yes

**Table 2.** Participant preferences and corresponding OAC-D modifications

Item preferences	Participant rationale	OAC-D modification
My life lacked a sense of purpose. I didn’t have a sense of purpose.	Participants thought the first was “less harsh” and the second was too “absolute.”	Option 1 was retained, Option 2 was eliminated
The things I did were not valuable. The things I did were not worthwhile.	Participants felt “worthwhile” was more personal while “valuable” was more outside of one’s self or external.	Option 1 was eliminated, Option 2 was retained
I am easily irritated. I am irritable.	Participants felt Option 1 was more situational vs. Option 2 which was more consistent with a static personality trait.	Option 1 was retained, Option 2 was eliminated

## Discussion

This cognitive interviewing study sought to obtain patient-level feedback on the appropriateness, comprehensibility, and overall acceptability of the draft OAC-D items. Participants expressed consistent preferences for the layout and response option format of the measure, and overall did not express concern or confusion about any of the item content. Querying about item-level preferences verified that the wording of items was understood as the authors intended and facilitated elimination of redundant items. Participants reported that the measure was easy to complete and accurately captured their experiences with depressive symptoms with no major concerns. Participants’ acceptance and approval of item content provides further support for utilizing items beyond the traditional DSM depression criteria to reflect their symptoms.

The present study highlights the importance of cognitive interviewing and incorporating patient perceptions into measure development (Willis GB 2005). While expert opinion is a critical element of scale development, only patients can describe their true experiences. Ignoring their perspectives not only minimizes the importance of their firsthand experience, but also increases the likelihood of developing a measure that misses patients who may be experiencing significant and impairing symptoms. This is particularly important for older adults, who often identify and experience constructs in unique developmental ways that are not accurately captured by legacy measures developed without patient input and a “one size fits all” intention.

This study has several limitations that will be important to address in the next phases of this research. First, participants were drawn from a single cancer center and the sample was homogeneous with respect to race and ethnicity, limiting its generalizability. Future evaluations of the OAC-D will include more purposive sampling to ensure diversity and that the scale can accurately capture different groups’ experiences. Similarly, these interviews were only conducted in English at this time as the scale will initially be developed in English. However, future studies seeking to expand the reach of the OAC-D should include cognitive interviewing with other language groups to ensure that the language and constructs are culturally valid and sensitive for capturing symptoms of those who speak other languages.

In sum, the present cognitive interview study provides important patient-level stakeholder feedback on the wording and format of the OAC-D scale. Refinements made to the items will be instrumental in enhancing its readability, acceptability, and ultimately, its accuracy in capturing the lived experiences of OACs with depression. In the next phase of this planned program of research, the revised OAC-D will be piloted in a small validation study to determine its preliminary psychometric properties.
